# ^13^C Metabolic Flux Analysis of Enhanced Lipid Accumulation Modulated by Ethanolamine in *Crypthecodinium cohnii*

**DOI:** 10.3389/fmicb.2018.00956

**Published:** 2018-05-15

**Authors:** Jinyu Cui, Jinjin Diao, Tao Sun, Mengliang Shi, Liangsen Liu, Fangzhong Wang, Lei Chen, Weiwen Zhang

**Affiliations:** ^1^Laboratory of Synthetic Microbiology, School of Chemical Engineering & Technology, Tianjin University, Tianjin, China; ^2^Key Laboratory of Systems Bioengineering, Ministry of Education of China, Tianjin, China; ^3^Collaborative Innovation Center of Chemical Science and Engineering, Tianjin, China; ^4^Center for Biosafety Research and Strategy, Tianjin University, Tianjin, China

**Keywords:** metabolic flux analysis, chemical modulators, ethanolamine, lipid accumulation, NADP^+^-dependent malic enzyme, *Crypthecodinium cohnii*

## Abstract

The heterotrophic microalga *Crypthecodinium cohnii* has attracted considerable attention due to its capability of accumulating lipids with a high fraction of docosahexaenoic acid (DHA). In our previous study, ethanolamine (ETA) was identified as an effective chemical modulator for lipid accumulation in *C. cohnii*. In this study, to gain a better understanding of the lipid metabolism and mechanism for the positive effects of modulator ETA, metabolic flux analysis was performed using ^13^C-labeled glucose with and without 1 mM ETA modulator. The analysis of flux distribution showed that with the addition of ETA, flux in glycolysis pathway and citrate pyruvate cycle was strengthened while flux in pentose phosphate pathway was decreased. In addition, flux in TCA cycle was slightly decreased compared with the control without ETA. The enzyme activity of malic enzyme (ME) was significantly increased, suggesting that NADP^+^-dependent ME might be the major source of NADPH for lipid accumulation. The flux information obtained by this study could be valuable for the further efforts in improving lipid accumulation and DHA production in *C. cohnii*.

## Introduction

Docosahexaenoic acid (DHA) is a polyunsaturated fatty acid (PUFA) belonging to the ω-3 group. In recent years, DHA has attracted much attention because of its broad beneficial effects on human health (Glaser et al., 2011). As an important component in cellular membranes of human nervous, visual, and reproductive tissues, DHA is considered essential for the neurological development of infants. In addition, DHA plays significant roles in alleviating cardiovascular diseases, hypertension, diabetes, and neuropsychiatric disorders (Karr et al., 2011; Chitranjali et al., 2015). Therefore, DHA has been widely used in food, pharmaceutical and feed industries. The traditional source of DHA is fish oil, as ocean fish can accumulate ω-3 PUFAs by consuming DHA-rich algae as food (Ward and Singh, 2005; Walsh et al., 2016). However, the use of fish oil as a food additive is limited due to problems associated with its typical fishy smell, unpleasant taste, poor oxidative stability and difficult purification (Hajjaji et al., 2011). It is thus necessary to develop alternative approaches for commercial DHA production directly using marine DHA-producing microorganisms (Ganuza et al., 2008; Mendes et al., 2008; Ji et al., 2015). Efforts to explore microalgae as an alternative source of DHA have been made in recent years, such as adaptive evolution, strain improvement by mutation, and culture condition optimization (Guo et al., 2016, 2017; Sun et al., 2016, 2018; Liu et al., 2017; Ren et al., 2017).

*Crypthecodinium cohnii*, a flagellated marine microalga, has been considered as a prolific producer of DHA, which contains 25–60% DHA while less than 1% of other types of PUFAs in its total fatty acids (TFAs) (Pei et al., 2017). However, up to now, no genome sequence is available for *C. cohnii*, and the DHA biosynthetic pathway in *C. cohnii* remains elusive (de Swaaf et al., 2003; Mendes et al., 2008). Previous studies suggested that *C. cohnii* might utilize the polyketide synthases (PKS) route for the biosynthesis and accumulation of DHA, which requires no oxygen or NADPH-dependent desaturases (Ratledge, 2004). Meanwhile, Pei et al. (2017) recently applied *de novo* transcriptome analysis to characterize central carbohydrate and fatty acid biosynthesis in *C. cohnii*, which suggested that *C. cohnii* might utilize a combination of PKS systems and desaturase steps for DHA biosynthesis. In addition, metabolomics analysis has also been employed to understand the possible mechanisms responsible for the increased lipid accumulation (Li et al., 2015). These studies have broadened our understanding of the molecular and biochemical mechanisms underlying lipid accumulation in *C. cohnii*. However, until now the knowledge of the *C. cohnii* metabolic network is still very limiting due to lack of quantitative analysis of metabolic fluxes. Thus, it is urgently required to develop methodologies of quantitative metabolic analysis of *C. cohnii* in order to better understand the intracellular distribution of carbon fluxes as well as fluxes in response to extracellular stimuli. On the other hand, many efforts have been made to improve the production of lipids and DHA by *C. cohnii*, which can be achieved by the application of mutation, culture condition optimization and chemical modulators (Sijtsma and de Swaaf, 2004; Li et al., 2015; Liu et al., 2017). For example, early studies showed that antioxidants, such as sesamol had great potential to enhance cell growth and the biosynthesis of lipids in *C. cohnii* (Liu et al., 2015, 2017). Our previous study showed that ethanolamine (ETA) as chemical modulator, could increase lipid accumulation in *C. cohnii* by 18.78% (Li et al., 2015). Meanwhile, another study also showed that ETA was able to enhance lipid accumulation in *Scenedesmus obliquus* by 22% and has been considered as a potential inducer for improving lipid accumulation in model photosynthetic organisms (Cheng et al., 2012). Therefore, it will be valuable to determine the mechanism of ETA to modulate the lipid synthesis and promote the future modification on lipid accumulation and DHA production in the industry-important *C. cohnii*.

^13^C-labeling based metabolic flux analysis as an integrated experimental and computational method is an important approach to determine the dynamics of biochemical networks and to provide quantitative insights into the *in vivo* distribution of molecular fluxes throughout central metabolism (Zamboni et al., 2009). Recent studies showed that ^13^C-labeled metabolic flux analysis (^13^C-MFA) could be a powerful analytical technology for understanding lipid accumulation mechanisms in various oleaginous microorganisms, such as oleaginous microalga *Chlorella protothecoides* (Xiong et al., 2010; Zhao et al., 2015), oleaginous yeast *Yarrowia lipolytica* and *Trichosporon cutaneum* (Liu et al., 2013, 2016; Wasylenko et al., 2015; Zhang et al., 2016), and oleaginous fungus *Mucor circinelloides* (Zhao et al., 2015). However, no report is available on ^13^C-MFA in *C. cohnii* up to now. In order to systematically understand the mechanisms underlying lipid accumulation as well as quantitative metabolic information in *C. cohnii*, in this study, we utilized gas chromatography-mass spectrometry to analyze the ^13^C labeling patterns of the amino acids in biomass hydrolysates of *C. cohnii* grown in an optimized chemically defined medium with and without 1 mM ETA addition. By integrating these labeling measurement data with metabolite balancing, the intracellular flux distributions in *C. cohnii* were further quantitated. The study provided valuable information to promote the future modification on lipid accumulation and DHA production in *C. cohnii.*

## Materials and Methods

### Strain and Growth Conditions

*Crypthecodinium cohnii* ATCC 30556 was obtained from American Type Culture Collection (ATCC), and grown on chemically defined medium composed of (g/L): glucose, 9; K_2_HPO_4_, 0.1; MgCl_2_.6H_2_O, 10.6; CaCl_2_, 1.1; KCl, 0.7; Na_2_SO_4_, 3.9; SrCl_2_.6H_2_O, 0.1; KBr, 0.1; NaCl, 23.5; NaHCO_3_, 0.2; disodium glycerophosphate, 0.15; 3 mL Tris buffer; 5 mL of metal mixture; 1 mL vitamin solution and nitrogen source. Metal mixture composed of (g/L): FeCl_3_.6H_2_O, 0.5; Na_2_EDTA, 10; H_3_BO_3_, 10; CoCl_2_.6H_2_O, 0.01; MnCl_2∘_4H_2_O, 1.6; ZnCl_2_, 0.1. The vitamin solution composed of (mg/L): biotin, 3; and thiamine, 100. The different inorganic nitrogen sources at a final concentration of 36 mM including NH_4_Cl, (NH_4_)_2_SO_4_, NaNO_3_, Ca(NO_3_)_2_, and KNO_3_. The organic nitrogen sources were 2 g/L yeast exact and various concentration of glutamate (0.5–5 g/L). The culture conditions of propagation were the same as those used in our previous study (Li et al., 2015). Briefly, the cells were grown in 250 mL Erlenmeyer flasks each containing 50 mL of medium. Cultures were maintained at 25°C and incubated in a reciprocal shaker shifting statically at 180 rpm. ETA was added at 36 h when the culture entering the early exponential phase and each concentration experiment was carried out in triplicate. Carbon isotope: [U-^13^C] glucose was purchased from Cambridge Isotope Laboratories, Inc. (>98%, Cambridge Isotope Laboratories, Inc., Andover, MA, United States). Labeling experiment: the carbon source was 20% [U-^13^C] glucose/L and 80% unlabeled glucose/L. ETA were purchased from Sigma (St. Louis, MO, United States) and other reagents used in the study were purchased from Sinopharm Chemical Reagent Co., Ltd., China.

### Determination of Physiological Parameters

Cell density was measured on an ELx808 Absorbance Microplate Reader (BioTek, Winooski, VT, United States) at OD_490_. For the determination of dry cell weight (DW), triplicate samples of the culture were collected, washed with double-distilled water and freeze dried overnight. The cell density corresponded to OD_490_ by the regression equation *y* = 1.941*x*-0.178 (*r*^2^ = 0.9988, *p* < 0.05), where *y* is the cell density (g dry cell weight L^-1^) and *x* is the absorbance of the cell suspension at 490 nm. The specific growth rate (μ) in the log phase was calculated by using the equation μ = (ln *X*_2_-ln *X*_1_)/(*t*_2_-*t*_1_), where *X*_1_ and *X*_2_ are the cell density at OD_490_ at time *t*_1_ and *t*_2_, respectively. The glucose concentrations in the culture supernatants were determined according to the glucose oxidase method as previously described (de Swaaf et al., 1999). To determine the macromolecular composition of *C. cohnii*, the Lowry method (Holdsworth et al., 1988) was used to measure protein content, and amino acid composition was obtained with an Amino Acid Analyzer (L-8900, Hitachi, Tokyo, Japan). The phenol-sulfuric acid method was used to determine intracellular carbohydrate and starch contents (Masuko et al., 2005). The KOH/UV method (Benthin et al., 1991) and the modified Schneider method (Herbert et al., 1971) were used to determine RNA and DNA concentrations, respectively. The total lipids were extracted using a previous method as described below (Yang et al., 2009). A statistical *t*-test model was applied for the comparative analysis, and *p*-value less than 0.05 were considered statistically significant.

### Total Lipid Extraction and Lipid Profile Analysis

Two methods were used to determine the lipid accumulation in *C. cohnii* cells. The first protocol involves fluorescence intensity measurements with excitation and emission wavelengths of 510 and 585 nm after Nile Red staining. A fluorescence spectrophotometer (F-2700FL, Hitachi, Tokyo, Japan) was used for the assay (Sui et al., 2014). The second protocol involves direct lipid extraction using a modified method described previously (Yang et al., 2009). Briefly, *C. cohnii* cells were collected at 60 h by centrifugation (3550 × *g*) for 5 min and freeze-dried to generate a lyophilized algal powder. 15–25 mg of lyophilized algal powder was used for extraction using a chloroform-methanol solution (2:1, v/v) with 0.01% butylated hydroxytoluene. The extraction process was repeated three to four times. The above extracts were washed with 1.0 mL of 1.0 M KCl followed by 1.0 mL of double-distilled water. The solvents were removed using a vacuum concentrator system (ZLS-1, Hunan, China). The lipid profile was analyzed using an Agilent 5975 MSD/7890 instrument (Agilent Corp., Santa Clara, CA, United States) according to previous publications (Xiong et al., 2008; Pei et al., 2017).

### GC-MS Analyses of Protein Hydrolysates

To ensure the cells are being cultured under steady-state conditions, the cells were harvested at 60 h by centrifugation (15,800 × *g*, 4°C) for 2 min. Proteinogenic amino acid preparation for GC-MS analysis was performed following standard protocols (Zamboni et al., 2009). Approximately 25 mg of lyophilized biomass was hydrolyzed with 2 mL of 6 M HCl at 110°C for 24 h. The hydrolysate was dried in an oven at 80°C for 12 h. The hydrolysate was re-suspended in water-free dimethylformamide (DMF) and then centrifuged at 12000 × *g* for 10 min. The supernatant was added with 50 μL N-tert-butyldimethylsilyl-N-methyltrifluoroacetamide (TBDMS, Sigma-Aldrich, St. Louis, MO, United States) and derivatived at 85°C for 1 h. The derivatived samples were analyzed by GC-MS using an Agilent 5975 MSD/7890 instrument (Agilent Corp, Santa Clara, CA, United States). The column was a HP-5MS (Restek, Bellefonte, PA, United States). The oven temperature was initially held at 60°C for 2 min and reached 180°C at 5°C per min, then raised to 260°C at 10°C per min, and finally held at 260°C for 5 min.

### Flux Analysis

For metabolic flux ratio analysis, a mass isotopomer distribution vector, MDV_α_ [Eq. (1)], is assigned on the basis of well-developed mathematical methodology (Nanchen et al., 2007).

$${MDV}_{\alpha }=\left[\begin{array}{c} \left(m_{0}\right)\\ \left(m_{1}\right)\\ .\\ .\\ .\\ \left(m_{n}\right) \end{array}\right]\overset{n}{\underset{i=0}{\Sigma }}m_{i}$$

Where m_0_ is the fractional abundance of molecules with monoisotopic mass and m_i > 0_ is the abundance of fragments with molecules with higher masses. iMS2Flux software was used to correct the factional labeling distribution of the amino acids for natural isotopic abundance (Hart et al., 2012). The resulting MDV_α_values from iMS2Flux software are used to assess the fractional labeling (FL) enrichment of each fragment using Eq. (2).

$$FL=\frac{1}{n}.\overset{n}{\underset{i=1}{\Sigma }}m_{i}.i$$

Where *n* represents the number of amino acid carbon atoms in the considered fragment and *i* is the different mass isotopomers. OpenFLUX software was utilized under MATLAB environment (Mathworks, Inc., Massachusetts) to solve for the fluxes (Quek et al., 2009). The application is based on the elementary metabolite unit (EMU) framework (Antoniewicz et al., 2007). Stoichiometric data on growth, substrate uptake rate, storage formation, and on the cellular composition of *C. cohnii* together with mass isotopomer distribution data of the labeled amino acids that were produced using the iMS2Flux software were used as model input.

### Enzyme Activities Analysis

The cells were harvested at 60 h by centrifugation at 3000 × *g* for 5 min, and the pellet was resuspended in extraction buffer (containing 100 mM KH_2_PO_4_/KOH (pH 7.5), 20% (v/v) glycerol, 1 mM benzamidine⋅HCl, and 1 mM DTT). HNX-2 cell disruptor (Honour, Tianjin, China) was used to rupture cells. The supernatant was collected by centrifugation at 10,000 × *g*, 4°C for 15 min. The supernatant containing cytoplasmic and mitochondrial enzymes was subjected to the following enzyme activity analysis. The activities of NADP^+^-dependent ME and NADP^+^-dependent isocitrate dehydrogenase (ICDH) were determined using continuous spectrophotometric assays following the increase of NADPH (Hsu and Lardy, 1969; Liu et al., 2015; Safdar et al., 2017). The absorbance of the cuvettes at 340 nm was determined using a UV-1750 instrument (Shimadzu, Kyoto, Japan). The enzyme activity was defined as the reducing amount of NADP^+^ (nM) catalyzed by the enzyme solution with 1 mg of protein in 1 min (nM/min/mg protein). The negative controls were set as without the substrate (ME or isocitrate) and without the cell exact. The enzyme activity was normalized by negative controls. Standard Bradford method was used to determine protein concentration (Bradford, 1976).

### Statistical Analysis

In this study, each experiment was performed in three biological replicates. All data were reported as means ± standard deviations and were analyzed with a *t*-test.

## Results and Discussion

### Optimization of Chemically Defined Medium for ^13^C Metabolic Flux Analysis

**FIGURE 1:**
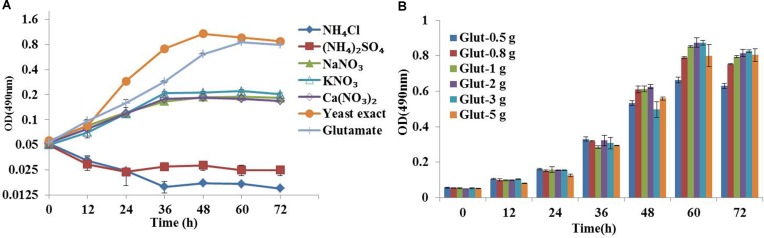
Optimization of chemically defined medium for ^13^C metabolic flux analysis. **(A)** Growth curves of *Crypthecodinium cohnii* under different inorganic nitrogen sources at a final concentration of 36 mM, 2 g/L yeast exact and glutamate. **(B)** Time courses of cell growth under different concentrations of glutamate (0.5, 0.8, 1, 2, 3, and 5 g/L).

Medium composition is a key factor for flux analysis (Zamboni et al., 2009). The yeast extract composition in natural medium is complicated and provided part of carbon source, which could increase the number of carbon substrates and might compromise flux calculability. Therefore, we first developed a synthetic medium suitable for ^13^C metabolic flux analysis based on the ATCC 460 A_2_E_6_ medium (Pleissner and Eriksen, 2012). In addition, the effects of five inorganic nitrogen sources (i.e., ammonium chloride, ammonium sulfate, sodium nitrate, potassium nitrate and calcium nitrate) and two organic nitrogen sources (i.e., glutamate and yeast exact) on *C. cohnii* growth were investigated in flask cultures. As shown in **Figure 1A**, the growth of *C. cohnii* in the inorganic nitrogen sources was significantly inhibited when compared to that in organic nitrogen sources, suggesting that the inorganic nitrogen sources were less efficient to promote cell growth. This was consistent with the previous study that *Chlorella protothecoides* grew slower in inorganic nitrogen sources than organic nitrogen sources (Xiong et al., 2008). The results showed that growth rates of *C. cohnii* were 0.051 and 0.054 h^-1^ in the medium supplied with 2 g/L glutamate and yeast extract, respectively, suggesting that *C. cohnii* was able to grow normally in the medium containing glutamate as nitrogen source. To further investigate the most optimal concentration of glutamate, different concentrations of glutamate ranging from 0.5 to 5 g/L were added. As shown in **Figure 1B**, no significant differences were observed when the concentration ranging from 0.8 to 2 g/L. However, obvious inhibition was observed when the concentration reached 3–5 g/L. Notably, high concentration of glutamate would dilute the ^13^C labeling substrate. Thus, 1 g/L of glutamate was chosen and used as the optimal nitrogen concentration in the following analysis.

### Influence of ETA Concentration on the Growth and Lipid Accumulation of *C. cohnii* in the Optimized Chemically Defined Medium

**FIGURE 2:**
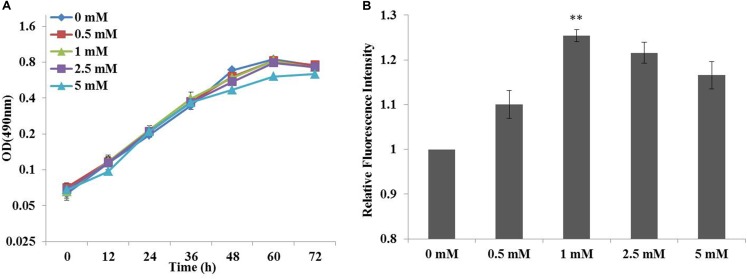
Effects of cell growth and lipid accumulation with ethanolamine (ETA) addition. **(A)** Growth curves of *C. cohnii* under different ETA concentrations. **(B)** The relative fluorescence intensity of *C. cohnii* stained with Nile Red after treated with different ETA concentrations. Statistical analysis was conducted as described in the text, as statistical significance indicated by ^∗∗^*p* < 0.01; ^∗^*p* < 0.05.

Previous studies had suggested that chemical modulators were able to enhance lipid accumulation in a diverse of microorganisms, and the roles of fourteen chemicals selected from five chemical groups had been established in *C. cohnii* (Li et al., 2015). Specifically, ETA as an amine could increase lipid accumulation in *C. cohnii* by 18.78%, which had the most significant effort on lipid accumulation among all chemical modulators evaluated so far. Here, influence of ETA concentration on the growth and lipid accumulation of *C. cohnii* in the optimized chemically defined medium was further investigated here. As shown in **Figure 2A**, the growth rates were 0.051, 0.048, 0.047, 0.043, and 0.035 h^-1^ in the optimized chemically defined medium with the addition of 0, 0.5, 1, 2.5 and 5 mM ETA, respectively. The results suggested that in this synthetic medium the growth rate was not significantly affected upon 0.5–5 mM ETA addition. Meanwhile, the neutral lipid content of *C. cohnii* supplemented with different concentrations of ETA was determined using the lipophilic stain Nile Red approach (**Figure 2B**). The results showed that the lipid content was increased by 25.4% with addition of 1 mM ETA compared with control, which was the most significant effect on lipid accumulation among all concentrations evaluated. Early studies on plant amine indicated that amines as cellular signals have an important role in metabolic regulation. In plants, the production of hydrogen peroxide (H_2_O_2_) deriving from amine oxidation has been correlated with cell wall maturation and reinforcement during pathogen invasion (Alcázar et al., 2010; Pál et al., 2015). Furthermore, increasing evidence indicated that reactive oxygen species (ROS) signaling might act as a mediator of lipid accumulation, which was associated with dramatic changes in the transcriptome, proteome, and metabolome in oleaginous microorganisms (Yilancioglu et al., 2014; Yu et al., 2015; Shi et al., 2017). In *C. cohnii*, it could also be assumed that ETA as a kind of signal molecule might cause the change of ROS which is responsible for the increase of lipid accumulation.

The growth characteristics of *C. cohnii* were then further determined in the optimized chemically defined medium. During the exponential growth phase, the specific growth rates of *C. cohnii* were 0.051 and 0.047 h^-1^ with and without 1 mM ETA (**Table 1**). The biomass yield didn’t show significant differences between both conditions while the glucose uptake rate with 1 mM ETA addition was slightly lower than the control.

**Table 1:** Growth parameters of *Crypthecodinium cohnii* in medium with and without 1 mM ethanolamine (ETA) addition^∗^.

ETA	μ	*Y*_X/S_	*q*_glc_
0 mM	0.051 ± 0.0002	0.388 ± 0.0075	0.657 ± 0.0556
1 mM	0.047 ± 0.0053	0.386 ± 0.0056	0.613 ± 0.0358


**^∗^μ, specific cell growth rate (h^-*1*^); Y_*X/S*_, biomass yield on glucose (g g^-*1*^); q_*glc*_, specific glucose consumption rate (mmol g^-*1*^ h^-*1*^)*.*

### Metabolic Model Construction and Biomass Composition Analysis

Based on the *de novo* transcriptome data obtained recently (Pei et al., 2017), we constructed a primary metabolic network of *C. cohnii*. As shown in Supplementary Table S1, central carbohydrate metabolism of *C. cohnii* includes the following core metabolic pathways: glycolysis pathway (EMP), pentose phosphate (PP) pathway, tricarboxylic acid (TCA) cycle and citrate pyruvate cycle (Pei et al., 2017). All involved reactions were assumed localized into two main compartments: cytosol and mitochondria, where most of the common metabolic reactions take place. All the biochemical reactions involved in the network are listed in Supplementary Table S1.

**FIGURE 3:**
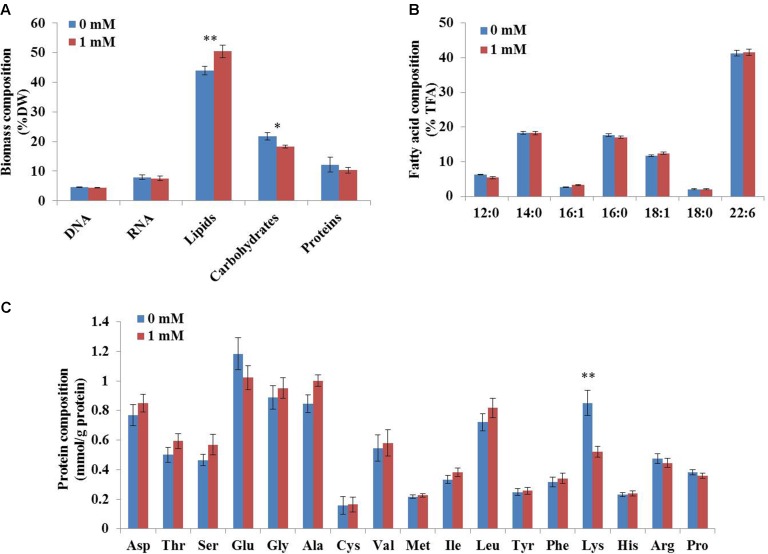
Biomass composition of *C. cohnii* with and without 1 mM ETA addition. **(A)** The fractional amounts of major biomass components. **(B)** The distribution of fatty acid composition. **(C)** The distribution of protein composition. Statistical significance was indicated by ^∗∗^*p* < 0.01; ^∗^*p* < 0.05.

To determine the coefficients in this biomass formation reaction, the biomass composition of *C. cohnii* was determined experimentally in this study. As shown in **Figure 3**, lipids were the most abundant component of *C. cohnii* (43.9% of dry weight), followed by carbohydrates (21.8%), proteins (12.8%), RNA (7.9%) and DNA (4.5%). The lipids content reached 50.45% in the presence of 1 mM ETA, which was 14.9% higher than the control culture, in accordance with the previous study that *C. cohnii* was treated with ETA on the medium consisted of glucose, yeast extract and sea salt (Li et al., 2015). The contents of carbohydrates and proteins were decreased by 20.7% and 18.1% in the presence of 1 mM ETA, respectively. Meanwhile, no significant difference of the DNA and RNA contents and the TFA content was observed between the cultures with and without 1 mM ETA. The relative distribution of amino acids in biomass was similar, with the notable exception of lysine, which was significantly lower with 1 mM ETA addition compared to the control culture (**Figure 3C**).

### ^13^C Metabolic Flux Analysis

**FIGURE 4:**
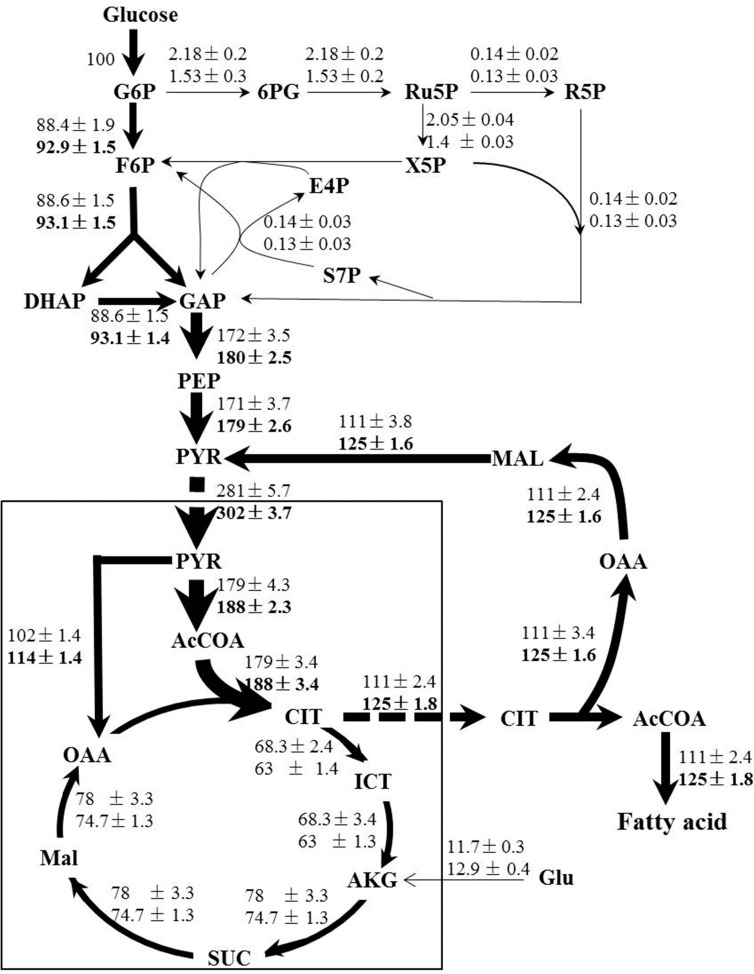
Flux maps of central carbon metabolism of *C. cohnii* in the presence of 0 and 1 mM ETA. The estimated fluxes were percentages of the relative rates normalized to the glucose uptake rates. The first and second line numbers represent relative flux when cultured without and with 1 mM ETA. The up-regulation fluxes with 1 mM ETA addition were in bold. G6P, glucose-6-phosphate; 6PG, 6-phospho-gluconate; Ru5P, ribulose 5-phosphate; F6P, fructose-6-phosphate; E4P, erythrose-4-phosphate; S7P, seduheptulose-7-phosphate; DHAP: dihydroxyacetone phosphate; GAP, glyceraldehyde 3-phosphate; PEP, phosphoenolpyruvate; PYR, pyruvate; AcCoA, acetyl-CoA; OAA, oxaloacetate; CIT, citrate; ICT, isocitrate; AKG, a-ketoglutarate; SUC, succinate; MAL, malate; Glu, glutmate.

Following the standard GC-MS measurement and amino acid biosynthesis schemes, the labeling patterns of both amino acids and their carbon backbone precursors in central carbohydrate metabolism were subsequently acquired. We then adopted a methodology named metabolic flux ratio analysis (Nanchen et al., 2007) to reveal directly the flux distribution of *C. cohnii* grown under the tested conditions. The estimated metabolic fluxes of *C. cohnii* with and without ETA were shown schematically in **Figure 4**. Fluxes were normalized to glucose uptake rate, which was given a value of 100%. As shown in **Figure 4**, in control culture, the carbon flux through the EMP and PP pathway accounted for 88.4 and 2.18% of the glucose uptake, suggesting that less carbon flux went through the PP pathway. As G-6-PDH and 6-phosphogluconate dehydrogenase in the PP pathway served as the primary alternative for producing NADPH (Ratledge, 2014), our results suggested that the PP pathway might not be the main way to supply NADPH for lipid biosynthesis, which was consistent with the previous study that no G-6-PDH activity was detected in any samples of *C. cohnii* (Liu et al., 2015). Most flux of the glyceraldehyde 3-phosphate was generated from the EMP. Pyruvate was synthesized by two different routes: pyruvate kinase and ME (Liu et al., 2013). Approximately 60.8% flux was generated from the EMP and 39.2% was generated from citrate pyruvate cycle, respectively. The citrate pyruvate cycle was responsible for conversion of pyruvate to oxaloacetate, catalyzed by pyruvate carboxylase. Then, oxaloacetate was converted to citrate, which was transported outside the mitochondria and degraded into oxaloacetate and acetyl-CoA in the cytoplasm. Malate obtained from oxaloacetate could then be decarboxylated to generate pyruvate via the NADP^+^-dependent cytosolic ME, playing an important role in lipid accumulation in oleaginous microorganism (Ronnebaum et al., 2006). About 63.7% flux of pyruvate was directed toward acetyl-CoA, and 36.3% was routed to the formation of oxaloacetate, respectively. In addition, the total flux through citrate was 179%, among which 62% flux of the citrate entered the lipogenesis pathway and 38% flux was catabolized through TCA cycle, respectively, suggesting that there were more flux through the lipogenesis pathway during lipid accumulation stage. In the TCA cycle, the flux through α-ketoglutarate was from iso-citrate (85%) and glutamate (15%), respectively. In this case, the results showed that glutamate was not only the nitrogen source, but also the carbon source. The fragments from proline, glutamate and aspartate were shown with low FL values (less than 0.15) (Supplementary Table S2), indicating that the labeling enrichments of these three amino acids were severely diluted by unlabeled glutamate in the medium. In order to avoid impairing accurate estimation of fluxes, the glutamate was set as a substrate during the flux analysis (Supplementary Table S1). The flux distribution obtained here provided quantitative metabolic information on *C. cohnii* and can be used to better elucidate and understand mechanisms involved in lipid metabolism.

When compared with condition of 1 mM ETA addition, our results showed that the flux ratio of EMP/PP pathway was increased to 92.9/0.53 when cultured with 1 mM ETA from 88.4/2.18 in control. The flux of EMP was increased by 5% and the flux in PP pathway was decreased by 29%, respectively. The results were in accordance with the previous metabolomics analysis, which suggested that the EMP metabolites were up-regulated and PP pathway metabolites were down-regulated, respectively (Li et al., 2015). Interestingly, the whole citrate pyruvate cycle was enhanced during lipid accumulation with 1 mM ETA addition. We thus speculated that the citrate pyruvate cycle might be enhanced by specific metabolic regulation that was triggered by ETA addition, which further promoted the lipid accumulation in *C. cohnii*. NADP^+^-dependent ME is located in cytoplasm and known to play a key role in lipid biosynthesis in oleaginous yeasts (Liu et al., 2013). Our results suggested that the flux through NADP^+^-dependent ME was increased by 12.6% with ETA addition. The results obtained in this study therefore pointed to the possibility that reaction via the cytoplasmic ME was the primary source to supply reducing equivalent for lipid biosynthesis in *C. cohnii*, which was in good agreement with several previous studies (Liu et al., 2015; Pei et al., 2017). For example, Liu et al. (2015) showed that *C. cohnii*, to a large degree, utilizes ME rather than ICDH or G-6-PDH to produce NADPH for the *de novo* fatty acid biosynthesis (Liu et al., 2015). In addition, the transcript of ME was found up-regulated 1.7-fold during lipid and DHA accumulation in *C. cohnii* (Pei et al., 2017). Furthermore, with 1 mM ETA addition, the flux of ICDH was decreased by 7.7%, suggesting that ICDH was slightly weakened, consistent with previous report that the activities of NADP^+^-ICDH and NAD^+^-ICDH in nitrogen-starved culture were reduced by 8 and 31%, respectively, which suggested that more carbon flux flowed to lipogenesis rather than to TCA cycle (Safdar et al., 2017).

### Activities of Key Enzymes

**FIGURE 5:**
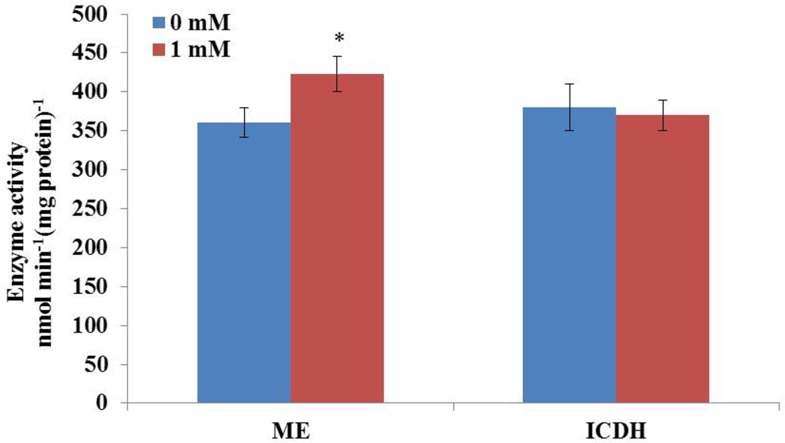
Effect of ETA on the activity of ME and IDCH of *C. cohnii* cultured with and without 1 mM ETA. Statistical significance was indicated by ^∗∗^*p* < 0.01; ^∗^*p* < 0.05.

It is well-known that the supply of reducing power in the form of NADPH plays an important role in fatty acid biosynthesis in oil-rich microorganisms. High content of lipid accumulation in *C. cohnii* requires enough supplies of acetyl-CoA as the precursor and reducing equivalent (NADPH) as the cofactor for fatty acid synthesis. In order to further confirm whether the activity of ME was increased upon 1 mM ETA addition, the activity of ME and ICDH were then measured. As shown in **Figure 5**, the activity of ME was 360.9 nmol/min/mg protein at 60 h with 1 mM ETA addition, which was increased by 17.2% compared with no 1 mM ETA addition. The result was consistent with the flux analysis result, which suggested that ME played an important role in lipid accumulation. Furthermore, the activity of ICDH was determined to be 370 nmol/min/mg protein at 60 h with 1 mM ETA addition, which didn’t have a significant change compared with no ETA addition. It was assumed that the ETA as a kind of signal molecule might cause the change of ROS responsible for the increase of lipid accumulation, which was still yet to be investigated and the improved ME productivity might contribute to the improved lipid accumulation. ME has been reported to be a major provider of the reducing power NADPH required for the lipid biosynthesis in oleaginous fungi (Hao et al., 2014). Overexpression of ME resulted in significant increase in lipid accumulation in yeast, fungi, microalga (Zhang et al., 2007; Li et al., 2013; Hao et al., 2014; Jiao et al., 2015). For example, overexpression of ME in *Mucor circinelloides* led to a 2.5-fold increase in lipid accumulation (Zhang et al., 2007). Heterologous expression of NADP^+^-dependent ME from *Mucor circinelloides* in oleaginous yeast *Rhodotorula glutinis* resulted in a 2.0-fold increase in lipid production (Li et al., 2013). Jiao et al. (2015) reported that the overexpression of ME (PtME) from *Phaeodactylum tricornutum* markedly increased the total lipid content in transgenic cells by 2.5-fold and reached a record of 57.8% increase of dry cell weight with a similar growth rate to wild type (Jiao et al., 2015). However, whether the improved ME activity was directly resulted from the addition of ETA, or by unknown secondary responses, was still yet to be determined. Collectively, it can be hypothesized that the flux through cytoplasmic ME might be associated with the lipid accumulation in *C. cohnii* and therefore, it could be a potential target for genetic modification to further improve the lipid content in *C. cohnii* in the future.

## Conclusion

In this study, the first ^13^C-metabolic flux analysis was performed in DHA-producing *C. cohnii*. Our results showed that with the addition of chemical modulator ETA, the flux through ME as well as the activity of ME were significantly increased, suggesting that NADP^+^-dependent ME might be the major source of NADPH for lipid accumulation. The analysis also suggested that in *C. cohnii* the whole citrate pyruvate cycle played an essential role in the lipid biosynthesis pathway. This study provided valuable information necessary for future genetic engineering of *C. cohnii* for improved lipid accumulation and DHA production.

## Author Contributions

LC and WZ conceived and designed the study. JC performed the experiments. JC, JD, TS, MS, LL, LC, and WZ analyzed the data and wrote the manuscript. All authors read and approved the manuscript.

## Conflict of Interest Statement

The authors declare that the research was conducted in the absence of any commercial or financial relationships that could be construed as a potential conflict of interest.
